# Sjögren’s Disease and Gastroesophageal Reflux Disease: What Is Their Evidence-Based Link?

**DOI:** 10.3390/medicina60111894

**Published:** 2024-11-18

**Authors:** Diana Mieliauskaitė, Vilius Kontenis

**Affiliations:** Department of Personalised Medicine, State Research Institute Center for Innovative Medicine, Santariskiu st. 5, LT-08405 Vilnius, Lithuania; vilius.kontenis@imcentras.lt

**Keywords:** Sjögren’s disease, gastroesophageal reflux disease, extraglandular manifestations, microbiota, Mendelian randomized study

## Abstract

Sjögren’s disease (SjD), or primary Sjögren’s syndrome (pSS), is a heterogeneous chronic autoimmune disorder with multiple clinical manifestations that can develop into non-Hodgkin’s lymphoma in mucosa-associated lymphoid tissue. SjD is one of the autoimmune diseases with the maximum delayed diagnosis due to its insidious onset, heterogeneous clinical features and varied course. It is increasingly recognized that extraglandular manifestations represent a clinical challenge for patients with SjD. The European League Against Rheumatism (EULAR) Sjögren’s Syndrome (SS) Disease Activity Index (ESSDAI) is a systemic disease activity index designed to measure disease activity in patients with primary Sjogren’s syndrome. It consists of 12 domains: cutaneous, pulmonary, renal, articular, muscular, peripheral nervous system, central nervous system, hematological, glandular, constitutional, lymphadenopathy and lymphoma, biological. More than a quarter of patients with pSS may have systemic features that are not included in the ESSDAI classification, i.e., various cardiovascular, ophthalmic, ENT, and other systemic or organ involvement that increase the magnitude of the systemic phenotype in the disease. The ESSDAI also excludes the gastrointestinal (GI) tract, and unfortunately, GI manifestations are not routinely assessed. Gastroesophageal reflux disease (GERD) is one of the most prevalent gastrointestinal disorders, impairing quality of life and consuming a large volume of medical resources. Recently carried out the Mendelian randomized trial confirmed the causal link between SjD and gastroesophageal reflux disease (GERD) and showed that GERD is a risk factor for SjD. This review aims to provide an overview of the research describing evidenced based links between Sjögren’s disease and gastroesophageal reflux disease, with the intention of ensuring that any systemic pathology in Sjögren’s disease is properly assessed and that management of the disease is directed towards the patient. A comprehensive literature search was carried out on PubMed, Web of Science, Scopus and the Cochrane Library databases. Two researchers searched for published studies indexed from inception to 1 September 2024 using the keywords ‘Sjögren’s syndrome’ OR ‘Sjögren’s disease’ AND ‘gastroesophageal reflux disease’ AND ‘microbiota’ OR microbiota dysbiosis’. We limited our search for scientific articles to human studies, and only included articles in English. Overall, there is a lack of evidence-based studies assessing the association between GERD and Sjögren’s disease and the changes in the microbiota associated with GERD in a multidisciplinary setting. Such studies are needed for the future, as this will improve the early diagnosis of Sjögren’s disease and the personalized management of the disease.

## 1. Introduction

Sjögren’s disease (SjD), or primary Sjögren’s syndrome (pSS), is a heterogeneous chronic autoimmune disorder with multiple clinical manifestations that can develop into non-Hodgkin’s lymphoma in mucosa-associated lymphoid tissue [1]. An analysis of the results from 62 studies provided an up-to-date summary of the available evidence on the incidence, prevalence, age of symptom onset and age of diagnosis of SjD globally, based on the 2016 ACR/EULAR diagnostic criteria. This summary identified a prevalence ranging from 12.4 to 13.1 per 100,000 person-years or 22.0 to 770.0 per 100,000 persons (once metrics were scaled to100,000 persons) [2]. SjD is one of the autoimmune diseases with the most delayed diagnoses due to its insidious onset, heterogeneous clinical features and varied course [3].

It is increasingly recognized that extraglandular manifestations represent a clinical challenge for patients with SjD, as the clinical features of the disease vary [4]. The European League Against Rheumatism (EULAR) Sjögren’s Syndrome (SS) Disease Activity Index (ESSDAI) is a systemic disease activity index designed to measure disease activity in patients with primary Sjogren’s syndrome. The ESSDAI is used as the gold standard for assessing disease activity in clinical trials. It consists of 12 domains (cutaneous, pulmonary, renal, articular, muscular, peripheral nervous system (PNS), central nervous system (CNS), hematological, glandular, constitutional, lymphadenopathy and lymphoma, biological) [5]. 

**Figure 1:**
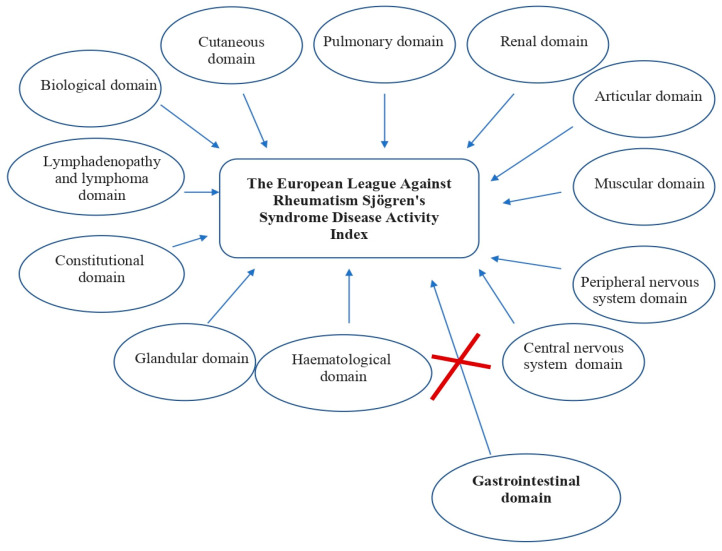
The European League Against Rheumatism (EULAR) Sjögren’s Syndrome (SS) Disease Activity Index (ESSDAI)—a systemic disease activity index designed to measure disease activity in patients with primary Sjogren’s syndrome.

EULAR Sjogren’s Syndrome Patient Reported Index (ESSPRI), and other patient-reported outcomes (PROs), such as the visual analog scale (VAS) for symptoms and EULAR sicca score (ESS), are used to assess the disease activity of primary Sjögren’s syndrome (pSS) [6]. More than a quarter of patients with pSS may have systemic features that are not included in the ESSDAI classification, i.e., various cardiovascular, ophthalmic, ENT and other systemic or organ involvement that increase the magnitude of the systemic phenotype in the disease. The ESSDAI also excludes the gastrointestinal tract [7]. Although the ESSDAI, ESSPRI and PROs do not include the gastrointestinal tract, the main gastrointestinal manifestations of Sjogren’s disease can occur in the esophagus, stomach, pancreas, liver and small intestine, and unfortunately are not routinely assessed [8,9] (Figure 1).

Gastroesophageal reflux disease (GERD) is one of the most prevalent gastrointestinal disorders, impairing quality of life and consuming a large volume of medical resources. Epidemiological studies show that the prevalence of GERD is steadily increasing worldwide. The prevalence of GERD ranges from 2.5% to 51.2%, depending on the population studied and the clinical features assessed, but the pooled prevalence of those studies that evaluated the criteria of weekly heartburn or regurgitation frequency was 13.3% (95% CI 12.0–14.6%) [10].

Notably, the just-published Mendelian randomized trial confirmed that there is a causal link between Sjögren’s disease and gastroesophageal reflux disease, and that GERD is a risk factor for SjD, while Sjögren’s disease itself has no effect on gastroesophageal reflux disease [11].

This review aims to provide an overview of the research describing evidenced based links between Sjögren’s disease and gastroesophageal reflux disease, with the intention of ensuring that any systemic pathology in Sjögren’s disease is properly assessed and that management of the disease is directed towards the patient. 

## 2. Materials and Methods

A comprehensive literature search was carried out on the PubMed, Web of Science, Scopus and the Cochrane Library databases. Two researchers searched for published studies indexed from inception to 1 September 2024 using the keywords ‘Sjögren’s syndrome’ OR ‘Sjögren’s disease’ AND ‘gastroesophageal reflux disease’ AND ‘gastrointestinal manifestations’ AND ‘microbiota’ OR microbiota dysbiosis’. We limited our search for scientific articles to human studies, and only included articles in English.

## 3. Sjogren’s Disease and Clinical Heterogeneity

The pathogenesis of SjD is multifactorial, resulting from the interaction between genetic factors and exogenous and endogenous factors, leading to an abnormal autoimmune response involving T and B lymphocytes. Inflammation maintains, perpetuates and enhances tissue damage, leading to progressive functional impairment of the affected organs [1,12]. SjD is a systemic disease that can affect almost all organs and systems. This clinical presentation is due to a variety of mechanisms: secondary exocrinopathy, autoimmune epithelitis with periepithelial lymphocytic infiltration of target organs, and organ autoimmunity with specific autoantibodies, and a systemic presentation involving immune complexes or cryoglobulinaemia and clonal lymphocyte expansion [12].

Patients with Sjögren’s disease are heterogeneous in terms of their clinical symptoms, systemic manifestations and risks. A recent study identified different groups of patients with Sjögren’s disease based on subjective symptoms, objective test results and biological parameters. The results of this study underline the fact that, even in patients with predominantly systemic symptoms, the symptom burden is high and should not be ignored. These results reinforce the need for appropriate assessment of patient complaints in all subgroups of Sjögren’s disease, irrespective of systemic activity [13].

### 3.1. Gastrointestinal Manifestations in Sjogren’s Disease 

Since 2015, the annual reviews of *Clinical and Experimental Rheumatology* have summarized the innovations in Sjögren’s disease research from that year. These annual reviews highlight the heterogeneity of the disease and the importance of the early detection of both glandular and extraglandular manifestations. Also, a recent systematic review highlighted the importance of early diagnosis of pSS, with recommendations for early recognition of the disease, particularly focusing on the recognition of organ-specific “hidden” signs of systemic disease. Although the annual *Clinical and Experimental Rheumatology* reviews aim to emphasize the importance of an early diagnosis of pSS, focusing on the recognition of organ-specific “hidden” signs of systemic disease, there are very few reviews of studies on the gastrointestinal manifestations of Sjogren’s disease, and no mention of GERD [14,15,16,17,18,19,20,21,22,23,24]. The 2018 review presents the results of a study showing that elevated levels of fecal calprotectin, a good marker of gastrointestinal inflammation, were found in a subgroup of patients with pSS and were associated with gastrointestinal tract comorbidities. Also, a study was reviewed in which severe intestinal dysbiosis was associated with clinical and laboratory signs of systemic disease activity, as well as with laboratory signs of gastrointestinal tract damage [18].

In a retrospective analysis of the Spanish Sjögrenser cohort, gastrointestinal involvement occurred in approximately 16% of patients, particularly as an autoimmune disorder, and in half of the patients, gastrointestinal involvement occurred at the same time as or after the diagnosis. Chronic atrophic gastritis was the most common form reported in approximately 30% of patients, followed by primary biliary cholangitis and autoimmune hepatitis. Pancreatic involvement was reported in 10% of patients and 7% had coeliac disease. Patients with gastrointestinal involvement were significantly older at the time of diagnosis of SS, were more likely to be female, and had a higher prevalence of autoimmune hypothyroidism and C3 hypo-complementemia [19].

In recent years, convincing evidence has suggested that patients with systemic rheumatic diseases, in particular pSS, may have an increased risk of coeliac disease (CD). A recent multicenter, case–control, Italian study involving more than 1400 patients with systemic autoimmune diseases, including pSS, systemic lupus erythematosus (SLE) and systemic sclerosis, reinforced this hypothesis. Primary SS patients were characterized by having a significantly higher prevalence of CD in comparison to a wide general population (6.78% vs. 0.64%). Moreover, pSS patients with CD were younger at autoimmune disease diagnosis in comparison to a nonceliac group, thus suggesting that screening for CD may be considered in young pSS patients, especially at disease diagnosis [21].

It was revealed that patients with pancreatic involvement had a higher prevalence of central nervous system and renal involvement, Raynaud’s phenomenon, lymphoma and hypocomplementemia in Sjogren’s disease [24].

#### 3.1.1. Gastroesophageal Reflux in Sjogren’s Disease

**Figure 2:**
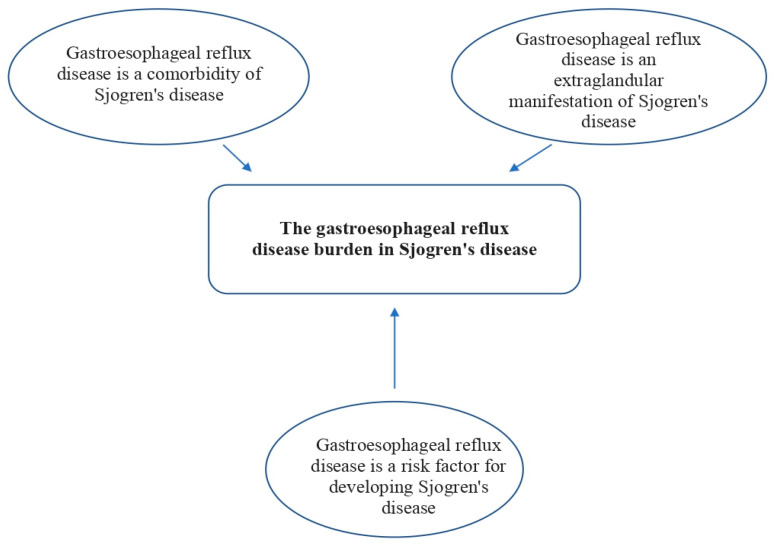
The association between Sjogren’s disease and gastroesophageal reflux disease.

As mentioned above, GERD was not mentioned when summarizing the results of the studies on gastrointestinal clinical manifestations in Sjogren’s disease in the annual reviews of *Clinical and Experimental Rheumatology*. However, to summarize other published studies on the association between Sjogren’s disease and GERD, they can be categorized as follows: GERD is a comorbidity of Sjogren’s disease; GERD is an extraglandular manifestation of Sjogren’s disease; and GERD is a risk factor for developing Sjogren’s disease (Figure 2).

The exact mechanism of the link between SS and GERD is not fully understood. Several explanations are given for why GERD occurs in people with SS. We know that the pathophysiology of GERD is multifactorial. A variety of factors can cause GERD symptoms, such as gastric anatomy and motility, the antireflux barrier, reflux properties, clearance mechanisms and mucosal integrity. On the one hand, studies have shown that the pathophysiology of the gastroesophageal tract of patients with SjD is different, and on the other hand, studies do not always exclude confounding factors (e.g., medication), which can lead to a bias in the results. Moreover, the sequence of the development of SS and GERD is not definitively clear [11,25,26].

##### Gastroesophageal Reflux Disease as a Comorbidity of Sjogren’s Disease 

In this section, we will review publications listing GERD as a comorbidity in Sjögren’s disease.

A Cox proportional hazards regression model was used to estimate the risk of gastroesophageal reflux disease in 4650 patients with Sjogren’s disease from 2000 to 2011. This study demonstrated that the risk of GERD among SS patients is 2.41-fold greater than that for general population [25]. A review of the literature summarizes the possible association between GERD and rheumatoid arthritis, mixed connective tissue disorders, Sjogren’s syndrome, systemic sclerosis and other diseases in which GERD is a comorbidity [26].

The results of the 2016 Sjögren’s Foundation survey, with 25 questions designed in a collaborative effort between the Foundation, patients with SjD, SjD provider experts and a marketing research company, revealed that 48 percent of SjD patients have had GERD as a medical comorbidity [27]. 

A recent study to determine how pain, dryness and fatigue—the main symptoms of Sjögren’s disease—contribute to cluster phenotypes identified GERD as a common comorbidity in Sjögren’s disease patients. The 1454 participants from the Sjögren’s International Collaborative Clinical Alliance (SICCA) Registry and 2920 participants of the Sjogren’s Foundation survey were divided into the following groups: (1) low symptom burden across all categories (LSB); (2) dryness with low pain and low fatigue (DLP); (3) dryness with high pain and low-to-moderate fatigue (DHP); and (4) high symptom burden across all categories (HSB). The Sjogren’s Foundation survey of 2920 people identified 1327 Sjogren’s disease patients with comorbid gastroesophageal reflux disease. A significantly high prevalence of GERD as a comorbidity was found in group DHP and the lowest in group LSB, respectively, out of groups LSB (*n* = 665), DLP (*n* = 409), DHP (*n* = 611) and HSB (*n* = 1235), with *n* = 237 (39%), *n* = 155 (41%), *n* = 288(49%) and *n* = 647(54%) [28]. 

More recently, a large sample study, one of the aims of which was to determine the relationship between the diagnosis of SD and the comorbidities diagnosed in these patients, found that 60% of patients with Sjögren’s disease reported oesophageal reflux as a comorbid condition [29].

The results of the study, which show that patients with Sjögren’s disease have a higher risk of gastroesophageal reflux disease compared to the general population, suggest that reflux is a common extraglandular manifestation of Sjögren’s disease [30]. The results of this study are flawed in that the prevalence may have been underestimated because only individuals who needed help were referred to the facility, the cross-sectional design used meant that a causal relationship based on the observed correlation between Sjögren’s syndrome and gastroesophageal reflux disease may not have been established. Medication use was not assessed in this study. Finally, as this study was conducted in a Taiwanese population, the results of the study are generalizable to other ethnic populations. All of these studies described above did not assess the effect of drugs on the development of GERD, which can be considered a limitation of these studies.

##### Gastroesophageal Reflux Disease as an Extraglandular Manifestation of Sjogren’s Disease

There is a dearth of scientific papers describing GERD as an extraglandular manifestation in Sjögren’s disease. An early study found no association between swallowing disorders and esophageal motor function in patients with Sjogren’s disease. The results of this study showed that pathological GERD, corresponding to the most common forms of dysmotility that are characteristic of GERD, was very common in SjD patients. These results underline the importance of an early and accurate diagnosis of GERD in SjD patients to prevent or correct esophageal pathology, as acid elimination from their esophagus is altered due to reduced saliva volume [30]. Also, another research study suggests that antibody-mediated disruption of M3-muscarinic receptor neurotransmission and the subsequent inhibition of smooth muscle contraction may lead to the gastrointestinal disturbances that are characteristic of Sjögren’s disease [31,32].

In a study published more than a decade ago to assess the prevalence of gastroesophageal reflux disease (GERD) symptoms and tooth wear in patients with Sjögren’s syndrome (SS) compared to controls, a statistically significantly higher proportion of SS patients reported suffering from heartburn and regurgitation compared to controls [33].

The review article highlights the problems that arise in the diagnosis of Sjogren’s disease and the prognosis of the outcome of the disease, mentioning gastroesophageal reflux as an extra-glandular manifestation [34]. 

Gastroesophageal reflux (GER) has also been shown to be common in Sjogren’s disease and can cause or contribute to a range of symptoms (such as heartburn, nausea, chronic cough, nausea, dysphagia). In this case–control study, the prevalence of GER was observed to be 60% in pSS patients compared to 23% in the control group [29]. A cross-sectional population-based study in Taiwan found that 4650 patients with SjD had a 2.4-fold higher risk of GERD compared to controls after adjusting for age, sex and comorbidities. Possible predictors of GERD in SjD may include reduced saliva volume, reduced esophageal motility, sphincter relaxation, slower gastric emptying time or side effects from medications [35].

Gastroesophageal reflux (GER) is present in 13 to 60% of pSS patients with gastrointestinal manifestations according to different researchers [7,36].

Chronic glandular inflammation impairs the protective function of the pharyngeal, laryngeal and esophageal mucosa against acid and gastric protease damage in Sjögren’s disease. Tissue immunity depends on the protective mucin cell layer, which weakens the diffusion of hydrochloric acid and changes the pH. When the epithelium is under immune attack and inflammation develops, the pharynx, larynx and esophagus are unable to react adequately to the damaging effects of gastric reflux [35].

A survey of people claiming to have primary and secondary Sjogren’s syndrome and sicca syndrome found that 86.97% of them experience intermittent/permanent dry mouth, and almost 80% of them experience GERD symptoms [37]. 

The study described in the previous section, in which the prevalence of GERD comorbidities was significantly highest in the dryness with high pain and low to moderate fatigue (DHP) group and lowest in the low symptom burden across all categories (dryness, pain, fatigue) (LSB) group [28], suggests that GERD is an extraglandular manifestation of Sjogren’s disease.

##### Gastroesophageal Reflux Disease as a Risk Factor for Developing Sjogren’s Disease: Insights from Mendelian Randomization Studies

There has been an observation that, in patients with Sjogren’s disease, the SjD regimen did not improve GERD-related symptoms, and in some patients, reflux was exacerbated due to side effects of the drug. Surprisingly and inexplicably, when patients with SjD received medication for GERD, their condition improved at the same time. It was hypothesized that there might be a genetic link between GERD and SjDS, and that GERD might influence the onset and course of SjD [10]. To date, until this Mendelian study, there had been no evidence-based research on the role of GERD in determining the risk of developing SjD. The Mendelian randomized trial confirmed that there is a causal link between Sjögren’s disease and gastroesophageal reflux disease, and that GERD is a risk factor for SjD, while Sjögren’s disease itself has no effect on gastroesophageal reflux disease. This study is based on clinical observations and the authors hope that the results will also be used for clinical diagnosis and treatment. The authors emphasize that we should pay attention to the likelihood of SjD when diagnosing GERD, and that physicians can alleviate GERD symptoms in SS patients to control the progression of SS [11]. 

This study has limitations due to the sample size and the fact that only European populations were selected for the study. In the future, the authors of the mentioned study plan to expand the sample size and sample information to include more different ethnic groups in order to confirm a causal link between GERD and SjD.

The results of another recent Mendelian study suggest that some microorganisms may have a protective role against the development GERD, such as the *Family Clostridiales Vadin* BB60 group, *Genus Lachnospiraceae UCG004*, *Genus Methanobrevibacter* and *Phylum Actinobacteria*, whereas other microorganisms, such as the *Class Mollicutes*, *Genus Anaerostipes* and *Phylum Tenericutes*, may be risk factors for the development of GERD. With regard to reverse causality, where GERD is a consequence and the gut microbiota is a result, the studies show that GERD is caused by dysbiosis in 13 different classes of gut microbiota. This Mendelian study has shown a genetic link between changes in the abundance of gut microbiota and the risk of GERD. This not only supports the feasibility of gut microenvironmental treatment of GERD, but also lays the groundwork for advanced research into the role of the gut microbiota in the etiology of GERD [38].

Recently, it has been shown that the gut microbiota, metabolites and metabolic pathways in healthy children and children with GERD are different, and that differences in metabolites are associated with specific changes in bacterial abundance. In the future, this may provide new evidence on the pathogenesis of GERD, and possibly expand our knowledge of Sjogren’s disease [38].

Helicobacter pylori is a Gram-positive bacterium and plays a role in the inflammation and carcinogenesis of the stomach lining. Helicobacter pylori infection is estimated to affect a large proportion of the world’s population, with a prevalence ranging from 60.3 to 80% [39,40,41,42,43,44,45,46,47,48]. The results of two Mendelian randomization analyses reveal a causal link between H. pylori infection and an increased susceptibility to GERD, and may lead to a better understanding of the pathogenesis of GERD [44]. 

A bidirectional Mendelian randomized trial found that GERD may increase the likelihood of insomnia, snoring and obstructive sleep apnea, in addition to shortening sleep duration [45], and undoubtedly contributes to the increase in oral dryness and chronic fatigue in patients with SjD and GERD.

##### Characteristic Changes in Microbiota in Sjogren’s Disease and Gastroesophageal Reflux Disease

GERD can increase the risk of SS due to changes in the gut and esophageal microbiota. A recent study shows that *Bacteroides* and *Prevotella* increase significantly in GERD patients, while *Actinobacteria*, *Lactobacilli, Micrococci*, *Rotella and Streptococci* decrease significantly in these patients [49]. Dysbiosis of the microbiota in the gastrointestinal tract due to gastroesophageal reflux disease may in turn increase the risk of Sjögren’s syndrome [38,39]. The esophageal microbiome of GERD patients and healthy individuals has been studied and is characterized by two main types: a type I microbiome, which is associated with a healthy state and is dominated by Gram-positive bacteria, and a type II microbiome, which is associated with GERD and is characterized by the pronounced prevalence of Gram-negative and microaerophilic bacteria [50]. Recent evidence suggests that GERD may develop through an immunogenic pathway. The evidence shows that submucosal inflammation induced by cytokines with intact epithelial cells is observed in the distal esophagus of patients with GERD, likely due to an immunogenic pathway [40,51]. 

A recent study using 16S ribosomal RNA (16S rRNA) gene sequencing analysis investigated the association between salivary microbiota and GERD. GERD patients had a higher relative abundance of *Bacteroidetes phylum, Bacteroidia* class, *Bacteroidales* order, *Prevotellaceae* family and unidentified_*Prevotellaceae* genus. A comparison with individuals with undiagnosed GERD revealed a reduction in the *Actinobacteria phylum*, the unidentified *Actinobacteria* and *Bacilli* classes, the *Micrococcales* and *Lactobacillales* orders, the *Micrococcaceae* and *Streptococcaceae* families and the genuses *Rothia* and *Streptococcus* in the saliva of patients with GERD [39]. Although the authors of this study claim to have identified changes in salivary microbiota and biomarkers in GERD patients, the study is flawed due to the small sample size, the lack of healthy controls and the lack of an analysis of the effect of ph on oral microbiota.

Another recent study has identified distinct microbiota populations in the distal esophagus associated with different stages of gastroesophageal reflux disease [52]. A new study by the same authors shows that the oral microbiome differs significantly between acid reflux severity groups. This study suggests that excessive gastric acid reflux into the esophagus may lead to disturbances in the homeostasis of the oral microbiota, suggesting that the oral ecosystem of patients with GERD will be altered as a direct result of the gastric reflux and the alteration of saliva production. However, the design of this study is limited because oral saliva pH was not measured [53].

A recent Mendelian randomization (MR) analysis revealed a possible causal effect of specific microbial taxa on functional dyspepsia (FD) and irritable bowel syndrome (IBS), the symptoms of which are often overlapping with those of gastroesophageal reflux disease (GERD) [54]. The study has limitations in that potential causal relationships may not have been explored for all species, the European population studied may limit generalizability to other populations, and the small sample size studied.

Regarding the role of the microbiota, there is a lack of studies that simultaneously investigate the expression of the microbiota at different sites in the gastrointestinal tract of patients with GERD, Sjögren’s disease. Further research into the links between GERD, Sjögren’s disease and the microbiota on appropriate design will help to elucidate the pathogenesis of Sjögren’s disease in the future, and will lead to the development of effective disease monitoring and treatment.

## 4. Conclusions

Overall, there is a lack of evidence-based studies assessing the association between GERD and Sjögren’s disease and the changes in the microbiota associated with GERD in a multidisciplinary setting. Such studies are needed for the future, as this will improve the early diagnosis of Sjögren’s disease and the personalized management of the disease.

## Data Availability

Publicly available datasets were analyzed in this study and referred to in the list of references.
